# The Not-So-Good, the Bad and the Ugly: HPV E5, E6 and E7 Oncoproteins in the Orchestration of Carcinogenesis

**DOI:** 10.3390/v13101892

**Published:** 2021-09-22

**Authors:** Om Basukala, Lawrence Banks

**Affiliations:** Tumour Virology Laboratory, International Centre for Genetic Engineering and Biotechnology, Padriciano 99, I-34149 Trieste, Italy; basukala@icgeb.org

**Keywords:** HPV, viral oncoproteins, E5, E6, E7, carcinogenesis

## Abstract

Infection with HPV starts with the access of the viral particles to basal cells in the epidermis, potentially via microtraumas to the skin. The basal cells are able to keep away these pathogens in normal circumstances through a robust immune response from the host, as HPV infections are, in general, cleared within 2 to 3 weeks. However, the rare instances of persistent infection and/or in cases where the host immune system is compromised are major risk factors for the development of lesions potentially leading to malignancy. Evolutionarily, obligatory pathogens such as HPVs would not be expected to risk exposing the host to lethal cancer, as this would entail challenging their own life cycle, but infection with these viruses is highly correlated with cancer and malignancy—as in cancer of the cervix, which is almost always associated with these viruses. Despite this key associative cause and the availability of very effective vaccines against these viruses, therapeutic interventions against HPV-induced cancers are still a challenge, indicating the need for focused translational research. In this review, we will consider the key roles that the viral proteins play in driving the host cells to carcinogenesis, mainly focusing on events orchestrated by early proteins E5, E6 and E7—the not-so-good, the bad and the ugly—and discuss and summarize the major events that lead to these viruses mechanistically corrupting cellular homeostasis, giving rise to cancer and malignancy.

Most human cancers are caused by agents that cause DNA damage and genomic instability, leading to the deregulation of cellular homeostasis. Over the period of this transformation, cancer cells acquire the major hallmarks of cancer and are able to sustain proliferative signalling, evade growth suppressors, resist cell death, enable replicative immortality, induce angiogenesis, activate invasion and metastasis, deregulate cellular energetics and metabolism and avoid immune destruction. The acquisition of these eight functional capabilities is primarily facilitated by two main traits of cancer—genome instability with consequent gene mutation and tumour-promoting inflammation (Figure 1) [1,2]. Among other causes (smoking, radiation, cancer-causing chemicals or carcinogens, hormones, chronic inflammation, etc.), infectious agents like hepatitis B virus, hepatitis C virus, human papillomavirus (HPV), Epstein-Barr virus, HIV-1, human T-cell lymphotrophic virus-1, Merkel cell polyomavirus, Kaposi’s sarcoma herpesvirus, *Helicobacter pylori*, *Schistosoma haematobium* and *Opisthorchiasis viverrini* are known to cause approximately 15% of human cancers [3,4]. While most viruses have evolved to use host cellular machinery for their life cycle, certain tumour viruses, such as HPVs, express viral oncogenes that directly contribute towards cellular transformation and cancers. Although this multistep process leading to a transformed cell phenotype is not a permissive event for the viral life cycle, these viruses play a significant role in development and progression towards cancer and malignancy.

HPVs cause almost one-third of the 15.4% human cancers attributable to carcinogenic infections [3]. Cervical cancer charts as the second-most common cancer in women aged 15–44 years and the fourth leading cause of female cancer worldwide, and cervical cancer is almost always associated with infection with HPVs [5]. Other anogenital cancers and an increasing number of head and neck cancers, including those of the oral cavity, oropharynx, sinus, tonsil and larynx, are also caused by these viruses. HPVs belong to the Papillomaviridae family; they are nonenveloped virions with a double-stranded DNA genome enclosed in an icosahedral capsid composed of major (L1) and minor (L2) structural proteins. The genome contains eight to nine ORFs, designated early (E1, E2, E4, E5, E6 and E7) and late (L1 and L2) proteins according to the time of expression after infection. Based on L1 gene sequences, HPVs are classified into alpha, beta, gamma, mu and nu genera. These viruses are mainly associated with the host epithelium, and those alpha-HPV types that infect the mucous membranes have been grouped into high- and low-risk types. Infection with low-risk types is characterized by benign lesions, whereas lesions caused by high-risk types may progress to cancer. Although over 200 HPV types have been identified, only a small group of specific types are known to cause cancer and are classified as high-risk (HPV-16, 18, 31, 33, 35, 39, 45, 51, 52, 56, 58, 59, 63, 73 and 82); probable high-risk types (26, 53 and 66) and low-risk (6, 11, 40, 42, 43, 44, 54, 61, 70, 81 and CP6108) by the International Agency for Research on Cancer [6]. In this review, we will discuss how expression of the viral proteins E5, E6 and E7 orchestrates the rewiring of cellular homeostasis, leading to the development and progression to cancer and malignancy.

## 1. Carcinogenic Orchestration by E5, E6 and E7

**Figure 2:**
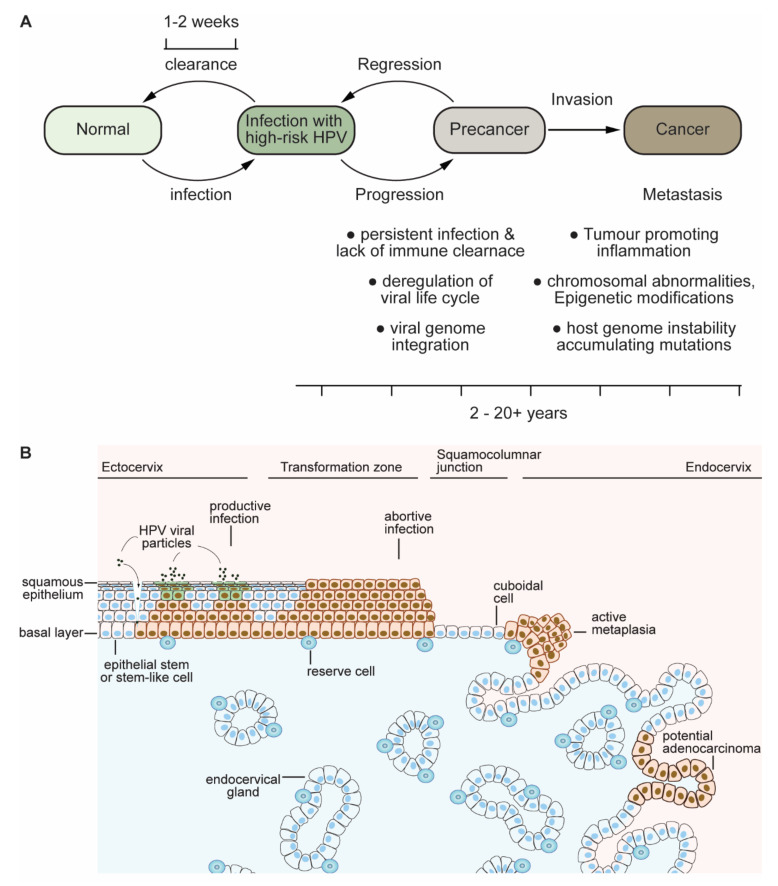
From infection to the development of cancer and malignancy, mediated by HPV (adapted and modified from References [22,23]. (**A**) Infection with HPV is normally cleared with 1 to 2 weeks; however, in certain individuals, persistent infection and a lack of immune clearance can lead to deregulation of the viral life cycle and viral genome integration, making them major risk factors for tumour development and progression towards cancer and malignancy. The progression towards invasive cancer and metastasis involves several changes, including chromosomal abnormalities, epigenetic modifications, genome instability and accumulating mutations and tumour-promoting inflammation, taking 2 to 20-plus years. (**B**) A cartoon representing infection by HPVs and progression to cancer. Infection with HPV is thought to occur via microtraumas in the epithelium, allowing access of the virus to the basal cell layer. HPV maintains its genome in the basal cells, and, as these cell divide, there is coordinated expression of early viral proteins, including E6 and E7, that allows the differentiation-determined cells to reinitiate the cell cycle. As these cells reach the upper squamous layers, with a concomitant expression of viral late gene products L1 and L2, new virions are released upon desquamation. Various regions of the cervix composed of stratified epithelium of the ectocervix, the transformation zone and the columnar epithelium of the cervix and endocervix are indicated in the cartoon, suggesting major sites of productive infections leading to the release of viral particles and abortive infection associated with deregulated HPV gene expression, potentially leading to squamous cell carcinoma and adenocarcinoma.

Infection with HPVs is believed to occur through contact with infected genital skin, mucous membranes or bodily fluids, and it can be sexually transmitted. Most (70–90%) of these infections are asymptomatic and are resolved by the host immune system within 1 to 2 years; however in some (5–10%) infected individuals, where the infection is not cleared, a persistent infection develops, which can ultimately lead to malignancy (Figure 2). Persistent infection with HR-HPVs may lead to inadvertent integration of the viral episomes into the host genome [7,8]. This event is not only unfortunate for the virus, as it can no longer complete its productive life cycle, but also to the host in promoting carcinogenesis [9]. Integration of the viral DNA often occurs at common fragile sites [10,11], while the event can also induce host genome rearrangements that could lead to direct activation/repression of oncogenes/tumour suppressors such as *myc* [12], perturbing cellular homeostasis. More than 70% of HPV-mediated cancers are caused by high-risk types 16 and 18 alone, and in the case of HPV-18-positive cancers, the HPV-18 genome is integrated in 100% of cases, whilst in the case of HPV-16, the virus can remain episomal in up to 25% of cancers [13,14,15]. In most cases, the integration event disrupts the repressive function of the E2 gene on the early promoter, leading to an overwhelming expression of E6 and E7, thereby rewiring cellular functions towards a carcinogenic fate [16]. Indeed, blocking the expression of E6 and E7 has been shown to inhibit tumour cell proliferation, leading to senescence and apoptosis [17,18,19]. Although the expression of E5 is mainly terminated by the integration event, the expression of E5 can play a significant role in promoting carcinogenesis by avoiding immune destruction. Furthermore, recent evidence in HPV-positive head and neck squamous cell carcinomas (HNSCC), where the integration of HPV-16 into the host genome is not often seen, suggests a dramatic increase in E5, together with E2 and E4, driving an alternative mechanism of carcinogenesis with a minimal expression of E6/E7 [20]. The concept of carcinogenic orchestration of HPV oncoprotein-mediated cancers is also backed by the fact that HPV-positive cancers have fewer somatic alterations and changes in the protein expression profile compared with HPV-negative cancers [21]. However, over the period of persistent infection, this continued genomic instability and evasion of immune destruction mediated by high-risk HPV oncoproteins results in an increasing accumulation of cancer-promoting host cell mutations, leading to cancer and malignancy.

### 1.1. E5, E6 and E7 Oncoproteins—An Overview

**Figure 3:**
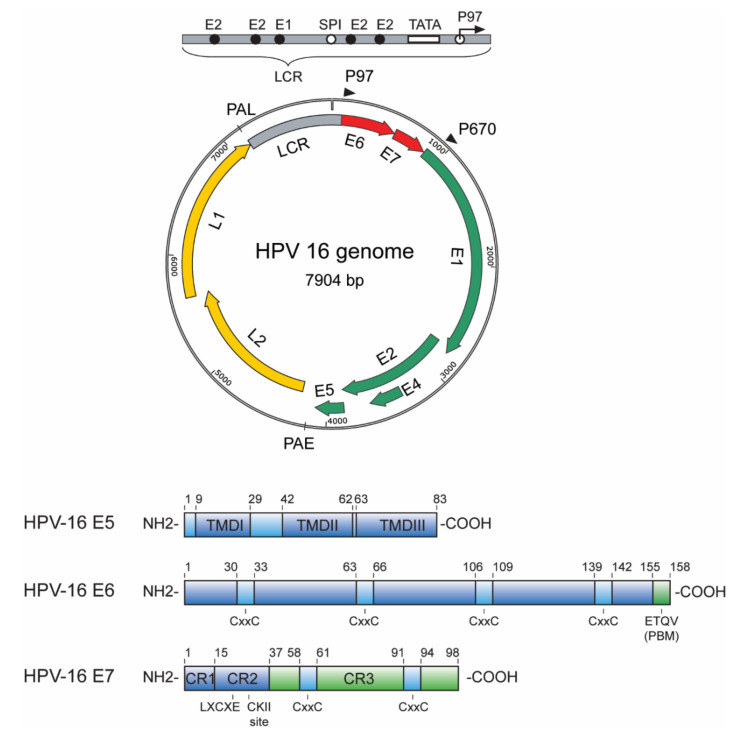
Schematic representation of HPV oncoproteins E5, E6 and E7. HPV16-E5 is an 83-amino acid-long transmembrane protein with three transmembrane domains (TMDI-III). HPV-16 E6 is an 158 amino acid protein with two CXXC (Cys-X-X-Cys,) motifs, which participate in coordinating with zinc ions and are indicated in the schematic. The C-terminus PDZ-binding motif (PBM) sequence ‘ETQV’ is shown, which can be phosphorylated by AKT, Chk2 via PKA and Chk1 kinases. HPV-16 E7 is a cytoplasmic phosphoprotein. The position of the conserved regions (CR1, CR2 and CR3) and CXXC (Cys-X-X-Cys) motifs, which participate in coordinating zinc ions, are also indicated. The LXCXE motif and CKII phosphorylation site in the CR2 region are important for targeting the pRB and related pocket proteins.

Among the five genera of HPVs, only the alpha HPVs encode and express E5. HPV E5s are small hydrophobic transmembrane proteins containing three hydrophobic transmembrane domains (TMD1-3), based on the molecular prediction and modelling analysis. A schematic structure of HPV-16 E5 is shown in Figure 3. HPV-16 E5 can self-associate both in vitro and in vivo and form oligomers by hydrophobic interactions [24,25,26]. Based on the biochemical characteristics and protein evolution, E5s are classified into E5, E5β, E5γ and E5δ, where those of high-risk HPVs fall in the E5α category and those of low risk fall in to the E5β, E5γ or E5δ families [27]. High-risk HPV-16 E5α is an 83 amino acid protein, localized mainly in the membranes of the endoplasmic reticulum, Golgi, nuclear membrane and endosomes and, also, in the plasma membrane [28,29]. Indeed, the presence of the E5 gene in the viral genome correlates with the carcinogenic potential [27,30].

The HPV E6 proteins are approximately 150 amino acids in length and are zinc-binding proteins with four CXXC motifs [31,32]. A schematic structure of HPV-16 E6 is shown in Figure 3. Both E6 and E7 are expressed from a common early promoter. E6 is transcribed either as a full-length (E6) mRNA or as one of several possible truncated E6 mRNAs (E6*) based on a complex splicing pattern. The E6* transcripts described in HPV-16 are E6*I, E6*II, E6*III, E6^E7, E6^E7*I, E6^E7*II, E6*IV, E6*V and E6*VI [33,34,35,36], while the four E6* transcripts that have been described for HPV-18 are E6*I, E6*II, E6*III and E6^E7 [37,38,39]. The complete crystal structure of E6, including the N-terminal and C-terminal halves, has been resolved, and a structure-functional analysis of E6 further suggests its plasticity in interacting with a wide range of cellular substrates [40,41,42,43]. The C-terminus of the high-risk E6 contains a unique signature sequence motif for interacting with PDZ (Post Synaptic Density 95 (PSD95), Discs Large (Dlg) and Zona Occludens 1 (ZO-1) proteins, a PDZ-binding motif (PBM)—and the presence of this motif has been suggested to correlate with oncogenicity, based on predictions of the array of PDZ proteins with which it interacts [43]. Furthermore, this motif can further be modified post-translationally (refer to review [44]) by phosphorylation by various kinases, altering its ability to interact with several of its targets and, hence, to modulate the host cellular functions [43,45,46,47,48,49].

HPV-16 E7 is a 98-amino acid-long heterogenous protein based on its structural and dynamic properties [50]. The spliced E6*I transcript has been suggested to be responsible for the translation of E7 [33]; however, recent evidence of circular RNA encompassing the E7 oncogene (circE7) has been demonstrated to make a significant contribution to the E7 protein levels and transforming properties, despite being a less abundant species (~1–3% of total E7 transcripts) [51]. The N-terminus of HPV16 E7 has sequence and functional homology to a portion of CR1 and to the entire CR2 region of adenovirus (Ad) E1a and related sequences in the simian vacuolating virus 40 large tumour antigen (SV40 TAg) [52,53,54]. A schematic representation of HPV-16 E7 is shown in Figure 3. The CR2 homology domain includes a LXCXE (X is any amino acid) motif, which is the interaction site for the retinoblastoma tumour suppressor (pRB) and related pocket proteins [55,56]; however, the optimum interaction requires the residues in the CR3 domain as well [57,58,59]. Adjacent to this motif is a consensus phosphorylation site for casein kinase II (CKII) at serines 31 and 32 in the case of HPV-16 E7 [60,61]. Additionally, in the C-terminus, the CR3 region contains two CXXC zinc-binding motifs separated by 29 amino acids [31,62]. Unlike the N-terminal part of E7, which is intrinsically disordered and is characterized by high flexibility [63,64,65], the C-terminus appears to be more structured and is also responsible for the formation of a homodimer, as shown by the 3D structure of the E7 CR3 regions from HPV1-A [66] and HPV-45 [64]. E7 has also been shown to form dimers [62,67,68], tetramers [69] and higher order oligomers [70,71]. HPV-16 E7 was shown to be a cytoplasmic phosphoprotein as early as 1987 [72]; however, nuclear pools have also been reported [73,74,75,76]. E7 has also been shown to be present in different subcellular (ER, Golgi and nucleus) compartments, based on immunofluorescence techniques using antibodies recognizing different epitopes in HPV-16 E7 [77]. Furthermore, E7 is post-translationally regulated by the proteasome and by phosphorylation (refer to review [44]).

### 1.2. Clues and Cues to Transformation and Cancer by HPV-Oncoproteins

The cellular transforming activity of high-risk HPV genomes was established in the mid-1980s in rodent cell line transformation assays [54,78], and subsequently, E7 was recognized as the major transforming protein of high-risk HPVs, using mutational analyses in transformation assays [52,79,80,81,82,83,84]. Later, using primary human keratinocytes, it was shown that high-risk HPV genomes cause lifespan extension, inhibit keratinocyte differentiation and lead to cellular immortalization [85,86,87]. Organotypic raft cultures expressing HPV genomes were also shown to have similar cellular alterations and abnormalities in tissue architecture as in high-grade HPV-associated clinical lesions [88,89]. Interestingly, HPV-16 E6/E7-expressing cell lines were immortalized but were not tumourigenic in nude mice, although they could induce tumours after several passages or in cooperation with additional oncogenes such as *ras* or *fos* [79,90,91,92]. A further mutational analysis showed that E7’s cooperation with E6 is necessary for these transforming activities in human keratinocytes, while such transforming and immortalizing activities are highly decreased in the case of low-risk E7s [93,94]. Similarly, co-transformation assays in murine kidney cells and human mammary epithelial cells established that HPV-16 E6 has transforming properties, inducing anchorage-independent growth and tumour formation in nude mice [95,96]; however, low-risk E6 were unable to do so [97]. Indeed, both E6 and E7 cooperate in transformation, where E7 drives the early tumourigenesis and E6 modulates the progression towards malignancy [90,98].

Several lines of evidence suggest that there is also a significant role of HPV E5 in contributing to oncogenic transformation. Early studies with BPV1 E5, the major transforming protein in BPV1 [99], led to studies of the oncogenic potential of the HPV E5 proteins in transformation assays. While HPV E5s display weak transforming activity in vitro in contrast to BPV E5, nonetheless, HPV E5 can transform mammalian cells. Initial studies with the expression of HPV-6 E5 in murine fibroblasts showed that it could induce the formation of colonies in soft agar [100], and later, HPV-16 E5 was also shown to induce anchorage-independent growth in murine keratinocytes and fibroblasts [101,102,103]. Further, HPV-16 E5 was shown to be tumourigenic in nude mice [102] and to cooperate with E6 and E7 in its transforming abilities, including the proliferation of primary rodent epithelial cells [104,105], immortalization of primary human keratinocytes [106] and enhanced migration and invasion in the human keratinocyte cell line [107,108]. In addition, studies in transgenic mice suggested that the expression of HPV-16 E5 in stratified squamous epithelia led to a higher frequency of spontaneous skin tumours [109] and caused cervical cancer when a prolonged oestrogen treatment was given [109,110]. As discussed earlier, upon integration of the HPV genome in high-risk HPV-18, E5 is often disrupted; however, in the case of HPV-16-positive cervical and oropharyngeal cancers, the expression of E5 is more likely and has been shown to be detectable [111,112,113,114,115], suggesting that E5 may contribute to malignant progression of the cancer.

## 2. Role Played by the HPV Oncoproteins towards Attaining the Cancer Hallmarks

Clues to the carcinogenic fate mediated by HPV oncoproteins, they have, for several decades now, led the search for mechanistic explanations, with the aim of determining possible strategies for therapeutic interventions, and many studies have demonstrated that several pathways are modulated by these oncoproteins in dysregulating normal cellular homeostasis. HPV oncoproteins do not have intrinsic enzymatic activity and nor do they share extensive sequence similarity with any host proteins [116,117], but they are able to interact with and modulate many host cell proteins to contribute to all the cancer cell hallmarks. Although most of these studies on the oncoproteins have been extensively reviewed previously, we will try to summarize and mention some of the key targets and pathways modulated by HPV oncoproteins during the process of carcinogenesis. Readers are recommended to refer to reviews on HPV oncoproteins and the related original articles for detailed descriptions [118,119,120].

### 2.1. Evading Growth Suppressors

The clonal regulation of human cells is unidirectional from stem cells to differentiated cells, unless they are induced by events of repair, crisis or external factors. In the case of the epidermis, the basal cells are responsible for continually replacing differentiated keratinocytes that safeguard the inner cells and tissues from external harm or pathogens. In epithelial differentiation, as the basal cell divides, the suprabasal daughter cell enters the differentiation pathway and withdraws from the cell cycle [121]. The viral life cycle is very closely linked with the epithelial differentiation program for the production, assembly and release of viral particles; however, the virus also inhibits differentiation to allow viral genome replication by inducing proliferation by targeting the pRB and p53 tumour suppressors. Several members of both the Rb and p53 pathways are classified as tumour suppressor genes, based on their frequent loss-of-function via deletion, intragenic mutations (e.g., p53 is mutated in almost 40% of all human cancers) or epigenetic alterations that compromise these tumour suppressor pathways. However, in the case of HPV-mediated cancers, both the p53 and pRB tumour suppressors are inactivated by the expression of E6 and E7, respectively. The expression of HPV-E7 can override the G1-S checkpoint to allow the cell to progress into the S phase by interacting with pocket family proteins (pRB, p130 and p107) and initiating E2F-dependent transcription. While the rescheduled DNA synthesis also allows activation of the p53 tumour suppressor, HPV-E6 can effectively inactive this response (see below). These two functions are the central players for the E6- and E7-mediated evasion of the cell’s growth suppressive functions. As discussed earlier, persistent infection and an inability to clear the infection by host immune response leads to integration of the viral DNA into the host genome, which, in turn, leads to a high-level expression of E6 and E7 and consequent promotion of the development of cancer and malignancy. Conversely, the silencing or repression of these oncoproteins activates senescence and apoptosis of the HPV-transformed cells [17,18,19,122,123].

**Figure 4:**
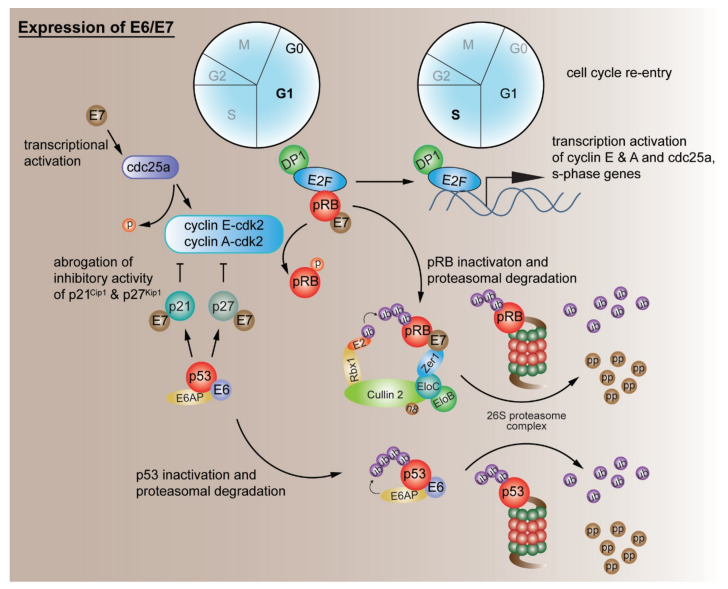
Evading growth suppressors. Unlike normal cells, where the growth and proliferation are tightly controlled, cells expressing HPV oncoproteins act to evade the key tumour suppressors pRB and p53. HPV-E7 can associate with pRB and inactivate the repressive function of the pRB/E2F transcription complex, leading to the expression of S-phase genes (cell cycle regulators, including cdc25a, cyclin E and A and replication enzymes and others) in otherwise cell cycle-exited and differentiating keratinocytes, leading to re-entry to the S phase. Further, high-risk E7 can downregulate pRB via proteasomal degradation via the cullin-2 ubiquitin ligase complex. HPV-16 E7 associates with cdk inhibitors p21^Cip1^ and p27^Kip1^, abrogates the inhibition of cdk2 activity and enhances the transcriptional activation of cdc25a, leading to the dephosphorylation of inhibitory phosphorylation in cdk-2/cyclin E/A. This leads to activation of the p53 tumour suppressor; however, high-risk E6 can inactivate and degrade p53 via the ubiquitin proteasome pathway involving the E6AP ubiquitin ligase complex. ub—ubiquitin and pp—2-25 residue peptides.

The pocket proteins and their interactions with members of the E2F family of transcription factors play key roles in regulating the cell cycle and apoptosis [124]. In normal cells, the association of pRB with E2F transcription factors prevents the transition of the G1/S checkpoint until the cell receives a signal to divide [125]. The G1-specific pRB/E2F transcriptional repressor complex is disrupted by the phosphorylation of pRB by cdk4/6 and cdk2 in late G1, and dissociated E2F acts as a transcriptional activator of the genes necessary for S-phase entry and progression. In HPV-infected cells, high-risk E7 can bind the G1-specific, E2F-bound pRB and disrupt this repressor complex, leading to uncontrolled G1 exit and S-phase entry [126]. E7 binds to pRB through the LXCXE motif in CR2, while the sequences in the E7 CR3 region have also been shown to be important [58,59]. In addition, high-risk HPV-16 E7 can destabilize pRB [127,128] through proteasomal degradation by interacting with the cullin 2 ubiquitin ligase complex [129] (Figure 4). Sequences in the E7 CR1 have also been shown to be necessary for pRB destabilization, in addition to the LXCXE-binding motif [128].

High-risk and low-risk E7s have differential abilities to bind members of the pRB family: low-risk E7 proteins bind to pRB with a much lower efficiency (approximately 10-fold less) than the high-risk HPV E7 proteins [55,130]. Again, only high-risk E7s can target all three of the pRB family proteins for degradation. Therefore, it has been suggested that the inability of low-risk HPV types to drive robust basal cell proliferation is because they can only efficiently induce the degradation of p130, which regulates cell cycle entry in the upper epithelial layers, but they cannot target p107 and p105, which regulate the cell cycle in the basal and parabasal layers [127,131,132,133,134,135,136,137].

#### G1/S Cell Cycle Checkpoint Deregulation by Other Mechanisms

In addition to the destabilization of pRB leading to perturbation of the pRB/E2F complex, E7 further enhances G1/S transition by binding to both positive and negative regulators of the cell cycle. Cyclin-dependent kinases (cdks) are drivers of the cell cycle. The regulatory subunits of cdk2, cyclins E and A, which drive S-phase entry and progression, are under the control of E2F, and it has been shown that cells expressing E7 maintain high levels of both cyclin E and A as a result of pRB destabilization and increased E2F-dependent transcription [138]. E7 can also directly associate with cdk2/cyclin A and cyclin E complexes, resulting in increased cdk2 activity [139,140,141]. Furthermore, HPV-16 E7 also causes an increase in the transcription of cdc25A (cell division cycle 25 A) phosphatase, which is involved in removing the inhibitory phosphorylation of cyclin E and A complexes, leading to the further activation of cdk2 activity [142]. More recently, E7-expressing cells were shown to have upregulated cdc6 [143], which promotes cell cycle progression by activating cdk2 [144,145], and furthermore, cdc6 was shown to be important in the G1/S transition in E7-expressing cells under hypoxic conditions [146].

HPV-16 E7 also associates with negative regulators of cell cycle and growth inhibitory activities. During keratinocyte differentiation, loss of contact with the basal membrane is accompanied by increased levels of cyclin-dependent kinase inhibitors (CKIs), subsequently inhibiting cdk2 activity and, thus, inducing a G1 growth arrest. However, HPV-16 E7 abrogates the inhibition of cdk2 activity by interacting with CKIs, p21^Cip1^ [147,148] and p27^Kip1^ [149], which are induced by antiproliferative signals, including growth factor withdrawal [150], activation of p53 [151] and loss of cellular adhesion [152,153]. Although E7 expression increases p21^Cip1^ through protein stabilization [154,155,156], cdk2 remains active in HPV-E7-expressing cells [147,148,157]. Furthermore, the abrogation of p21Cip1 inhibition has been shown to require C-terminus sequences of E7, where zinc-binding site mutants are shown to be proficient in targeting pocket proteins for degradation but are yet unable to overcome growth arrest [133,158]. Furthermore, this abrogation of p21Cip1 activity is less efficiently done by low-risk E7 [145,146]. Thus, the ability of HPV E7 to abrogate CKIs and disrupt pRB/E2F complexes, resulting in increased levels of cyclin A and E, establishes a replication-competent cellular milieu in differentiating keratinocytes, causing G1/S cell checkpoint deregulation that leads to continued proliferation.

Furthermore, HPV-16 E7 can target multiple members of the E2F transcription factor family, including the transcriptional activator, E2F1 and the transcriptional repressor E2F6 [159]. By interacting with E2F1, HPV-16 E7 can enhance E2F1-mediated transcription. E2F1 plays a role in mediating the transcriptional control of the E2F6 gene, which is upregulated in the G1/S-phase transition in order to repress E2F-responsive promoters, thereby checking the cell cycle for differentiation [160]. At the same time, HPV-16 E7 associates with E2F6 and perturbs its ability to function as a transcriptional repressor [161]. Together, these functions of E7 allow cells that are committed to exit the cell cycle and differentiation to remain in a S-phase-competent state, enabling them to initiate growth and proliferative signalling. However, the resulting unscheduled DNA replication also activates the cellular apoptotic pathways by a mechanism termed the ‘trophic sentinel response’ [162], but this is efficiently inactivated by high-risk E6 proteins (see below).

One of the most well-studied interactions of E6 is with the p53 tumour suppressor [163]. The p53 protein plays many roles in the cell, including cell cycle regulation, activation of DNA repair pathways upon DNA damage and induction of apoptosis [164]. By interacting with p53, E6 checks the transcriptional functions of p53, leading to the deregulation of p53-dependent gene expression [165]. In addition, high-risk E6 binds to an LXXLL motif on a cellular E3 ubiquitin-protein ligase, E6-associated protein (E6AP) [166,167,168,169], forming the E6-E6AP complex, which recruits and ubiquitinates p53, mediating its degradation via the proteasome [170,171,172] (Figure 4). The E6–E6AP complex has also been shown to be important for E6 stability, and the ablation of E6AP thus rescues p53 through two routes: as a result of E6 destabilization and the loss of E6AP ubiquitin ligase activity [173,174]. Furthermore, the interaction of E6 with E6AP has been shown to contribute to skin hyperplasia, spontaneous skin tumours and tumour progression in transgenic mouse studies, where mice harbouring a mutant HPV-16 E6, defective in binding E6AP, had greatly reduced E6-induced phenotypes [175,176]. Although a major part of E6 function has been shown to be associated with E6AP and its ligase activity, recent evidence suggests that E6 can also target some of its targets (MAGI-1 and Scrib) independently of the E6AP enzymatic activity [177].

The E6 interaction with p53 has been shown to perturb the binding of p53 to its site-specific DNA sequences [178], possibly as a result of conformational changes in the p53 protein upon interaction with E6, which, in turn, leads to an inhibition of the p53 DNA-binding ability [179]. Furthermore, E6 perturbs the p53 function by sequestering p53 in the cytoplasm, potentially by sterically hindering the p53 nuclear localization signal [180]. E6 has also been shown to abrogate the transactivation of p53-responsive genes via interactions with CBP/p300, [181,182,183], with hADA3 [184,185,186], and by destabilizing TIP60 [185]. Thus, by inactivating and perturbing several modulators of these tumour suppressor pathways, HPV-E6 and E7 are able to evade key growth suppressors of the cell. Evasion of the growth suppressors allows and ensures continued cell proliferation under circumstances where normal tissue homeostasis would have limited cellular proliferation. This, consequently promotes tumour growth and is one of the several hallmarks of cancer [2].

### 2.2. Resisting Cell Death

**Figure 5:**
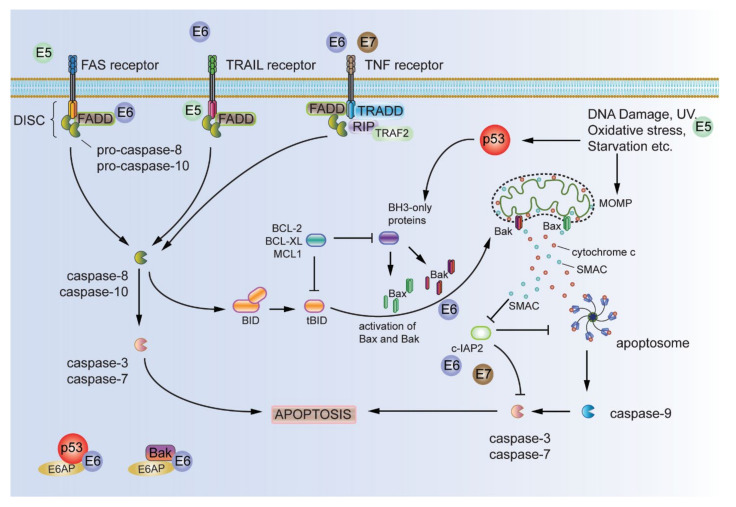
Resisting cell death. One of the major roles played by HPV oncoproteins in resisting cell death is evading apoptosis. Both extrinsic and intrinsic apoptotic pathways are deregulated to attain this function by HPV-E5, -E6 and -E7. The extrinsic apoptotic pathway is activated upon receptor trimerization and the subsequent recruitment of adaptor molecules and procaspase 8 to the DISC. The activation of caspase 8 then leads to the activation of downstream executioner caspases 3 and 7, leading to cell death/apoptosis. The intrinsic apoptotic pathway is activated by external stimuli (UV-radiation, oxidative stress, DNA damage, starvation, etc.), leading to the formation of pores in the mitochondrial membrane and release of mitochondrial inner membrane proteins (cytochrome c, SMAC) into the cytosol. Released cytochrome c and pro-caspase 9 form the apoptosome, leading to activating caspase 9, which, in turn, activates downstream executioner caspases 3 and 7, leading to apoptosis. E5 can downregulate the Fas receptor and perturb the formation of the DISC complex, thus abrogating the extrinsic apoptotic pathway. Further, E5 can perturb ROS-induced Bax activation and inhibit the apoptotic response to UV B radiation. E6 can block extrinsic pathways by binding the death domain, leading to its proteasomal degradation. E6 inactivates p53, Bax and Bak, thus abrogating MOMP and the release of cytochrome c and, thus, inhibiting the intrinsic apoptotic pathway. E6 can also inhibit antiapoptotic c-IAP2, blocking the formation of the apoptosome and activation of the executioner caspases. E7 seems to have a dual function in activating and abrogating apoptosis; however, E7 has been demonstrated to perturb TNF receptor-induced apoptosis by upregulating c-IAP2 and suppressing caspase 8. FADD—Fas-associated protein with death domain, TNF—tumour necrosis factor, TRAIL—FasL and TNF-related apoptosis-inducing ligand, TRAF2—TNF receptor-associated factor 2, DISC—death-induced signalling complex, c-IAP2—cellular inhibitor of apoptosis protein 2, RIP—receptor interacting protein and MOMP—mitochondrial outer membrane permeabilization.

Normal cells undergo apoptotic cell death upon extrinsic (e.g., the Fas ligand/Fas receptor) or intrinsic signals (e.g., radiation, toxins, hypoxia, etc. and, also, the release of proapoptotic signalling proteins, such as cytochrome c) and is one of the major cellular homeostatic programs to defy aberrant cell growth. The attenuation of apoptosis is one of the main hallmark features of cancers, and in the case of HPV infection, the infected cells should be doomed to undergo apoptosis due to cellular stress signals; however, HPVs have evolved potent mechanisms to avoid apoptotic cell death by abrogating multiple stages of the pathway. Modulation of the antiapoptotic functions by HPV is summarized in Figure 5.

In addition to inactivation of the p53-dependent apoptotic response (discussed above), E6 can further abrogate apoptotic signalling by interacting with the proapoptotic protein Bak and mediating its degradation via the E6AP ubiquitin ligase [187,188,189]. E6 was also shown to inhibit differentiation-induced apoptosis in human foreskin keratinocytes by modulating the expression of antiapoptotic Bcl-2 and proapoptotic Bax proteins [190]. Furthermore, the inhibition of E6 was shown to result in the p53-dependent transcriptional activation of the PUMA promoter, leading to the activation and translocation of Bax to the mitochondrial membrane, causing cytochrome c release into the cytosol and the activation of caspase 3. In addition, the inhibition of Bax expression in this context was shown to efficiently revert the apoptotic phenotype, suggesting that perturbation of the p53/PUMA/Bax cascade is an important antiapoptotic function of E6 in HPV-positive cancer cells [191].

Moreover, E6 interacts with the Fas-associated death domain (FADD) and procaspase 8 to enable cells to escape from Fas-triggered apoptosis [192,193]. In addition, E6 has been shown to evade apoptosis by downregulating the proapoptotic transforming growth factor-β2 (TGF-β2) and, thence, downregulating the TGF-β2 responsive gene expression [194]. Evidently, resisting cell death is efficiently coordinated by high-risk E6 by targeting various apoptotic pathways, which would otherwise have eliminated the HPV-infected cell. 

HPV-E5 also plays a significant role in resisting cell death by targeting several players of apoptotic signalling. HPV-16 E5 promotes the degradation of the proapoptotic Bcl-2 family member BAX upon oxidative stress [195]. The expression of HPV-16 E5 in primary human keratinocytes was also shown to defy ultraviolet (UV)-B radiation-mediated apoptosis. The attenuation of apoptosis in this case required E5-dependent EGFR activation, leading to enhancement of the PI3K-Akt and ERK1/2 MAPK signalling pathways [196]. Furthermore, the expression of HPV-16 E5 prevents FasL- or TNF-related apoptosis-inducing ligand (TRAIL)-mediated apoptosis by downregulating the expression of Fas receptors and abrogating the recruitment of the Fas-associated protein with death domain (FADD) to form the death-induced signalling complex (DISC) in raft cultures of HaCaT cells stably expressing HPV-16 E5 [197]. Thus, HPV-16 E5 also abrogates apoptosis by perturbing the regulators and effectors of apoptosis, contributing to the acquisition of carcinogenic hallmarks.

High-risk E7, on the other hand, has been shown to have mainly proapoptotic roles, rather than antiapoptotic roles; however, the expression of E6 efficiently counteracts the apoptotic signals induced by E7. One example of an antiapoptotic role of E7 is the ability of E7 to abrogate TNF-mediated apoptosis by obstructing the activation of pro-caspase 8 in E7-expressing human fibroblasts [198]. However, the major role of E7 in resisting cell death seems to lie in promoting the cell’s ability to survive and proliferate, even in the absence of adherence to an extracellular matrix (discussed below).

#### Resistance to Anoikis and Anchorage Independence

Anoikis is a form of apoptosis that is triggered in normal cells when they attempt to divide in the absence of a matrix [199]. p600 has been implicated in the regulation of anoikis signalling, and both high-risk and low-risk E7, as well as the E7 protein, from bovine papillomavirus 1 (BPV1), have been shown to associate with p600/UBR4 via residues in the CR1 amino-terminal domain [200,201]. The interaction of E7 and p600 has been shown to be necessary for the transforming ability of HPV-16 E7 [50,202]. In the case of BPV1 E7-expressing cells, the resistance to anoikis has been suggested to be the result of E7 expression [203]. More recently, the p600/UBR4 interaction with high-risk E7 was shown to be important for the destabilization of PTPN14 [204,205]. In PTPN14-knockout keratinocyte cell lines, the absence of PTPN14 expression was shown to be important for delaying differentiation in keratinocytes upon detachment from the basement. This phenocopies the effect of expressing high-risk HPV-16 E7, which inhibits differentiation upon cell detachment and allows cells to proliferate in suspension, suggesting that targeting PTPN14 by high-risk E7 through p600 allows E7-expressing cells to survive anoikis and proliferate, irrespective of their anchorage to the basement membrane [206,207], a characteristic hallmark of cancer cells in resisting cell death.

### 2.3. Sustaining Proliferative Signalling

One of the key roles that HPV E5 plays towards development of cancer is to thwart the activation of certain growth factor signalling pathways. These are tightly controlled in normal cellular homeostasis, but most cancer cells acquire the ability to enhance the proliferative signals relayed to cellular signalling pathways that regulate the cell cycle, cell growth, cell survival and energy metabolism, often through cell surface receptors typically containing intracellular tyrosine kinase domains. HPV-E5 has been shown to activate proliferative signalling in a number of ways. Early studies with EGF stimulation in cultured cells showed a transforming and mitogenic activity of HPV-16 E5 [101,102,208], and EGFR signalling was shown to be required for E5-induced epithelial hyperplasia in transgenic mice [109]. Indeed, several other studies have demonstrated elevated levels of EGFR at the cell surface upon HPV-16 E5 expression [101,207,208,209,210]. A mechanistic explanation of EGFR activation by HPV-E5 has suggested that it is dependent on the interaction of E5 with the vacuolar H^+^-ATPase (v-ATPase) abrogating endosomal acidification and reducing EGFR degradation upon EGF stimulation [211]; however, this has been challenged by other studies [212,213]. Furthermore, disruption of the E3 ligase c-Cbl and EGFR upon HPV-16 E5 expression was shown to result in a decrease in ubiquitination and degradation of EGFR, thereby enhancing EGFR-mediated mitogenic signalling [214]. Another growth factor signalling pathway that is affected by HPV-16 E5 is the G protein-coupled endothelin receptors (ET_A_) pathway. The mitogenic signalling of endothelin-1 (ET-1), a specific ligand of the G protein-coupled endothelin receptor (ET_A_), was demonstrated to be enhanced by HPV-16 E5 binding to ET_A_ in growth factor-starved keratinocytes, leading to keratinocyte proliferation, and this was suggested to potentially augment proliferative activity in conjunction with the EGFR pathways [215,216,217].

In addition, activation of EGFR signalling by HPV-16 E5 has also been linked to the activation of the c-Met growth factor receptor, a potent oncogene that contributes to the motility of HPV-containing cells [218]. Another epithelial receptor tyrosine kinase modulated by expression of HPV-16 E5 is the antiproliferative keratinocyte growth factor receptor/fibroblast growth factor receptor 2b (KGFR/FGFR2b). It is a major paracrine mediator of epithelial homeostasis and exerts a tumour-suppressive function. The expression of HPV-16 E5 downregulates the expression of KGFR2b, enhancing the aberrant expression of the mesenchymal FGFR2c isoform, which promotes the epithelial–mesenchymal transition (EMT), thus, in turn, potentially promoting malignant transformation [219,220]. Furthermore, HPV-16 E5 has been shown to activate mitogen-activated protein kinase (MAPK) p38 and ERK1/2 in human keratinocytes in an EGF-independent manner [221] via a receptor tyrosine kinase and protein kinase C (PKC) signalling pathway [222,223], ultimately leading to the increased transcription of transcriptional factor AP-1 (activating protein 1), which is composed of c-fos and c-jun and is responsible for promoting the cell cycle [224,225]. As HPV has an enhancer that contains AP-1-binding sites, this may further enhance the transcription of E6 and E7 oncogenes and, thus, contribute further to cellular transformation [105,225]. In addition to these growth control pathways, HPV-16 E5 also uses transcriptional and post-translational mechanisms, in both fibroblasts and keratinocytes, to downregulate the expression of p21^WAF1/CIP1^ and p27^KIP1^ cyclin-dependent protein kinase inhibitors, resulting in cell cycle progression and DNA synthesis [226,227]. Taken together, HPV-16 E5 can modulate several of the growth factor-mediated signalling pathways to promote a proliferative state in keratinocytes, thus contributing towards transformation, together with E6 and E7.

#### Modulation of Cellular Signalling Pathways

The E6 protein also modulates several survival pathways, including phosphoinositide 3-kinase (PI3K)/protein kinase B (AKT), Wnt and Notch. The PI3K/AKT pathway is a major cancer survival pathway regulating a broad range of downstream targets, including proliferation, cell growth, cell mobilization, angiogenesis and cell survival [228]. E6 has been shown to inactivate PTEN, leading to increased pAKT and increased cell proliferation [229]. The mammalian target of the rapamycin (mTOR) kinase, a downstream target of AKT, has been demonstrated to be activated by E6 via E6/E6AP-mediated degradation of the mTOR inhibitor tuberous sclerosis complex 2 (TSC2) [230,231]. In addition, under conditions of nutrient deprivation, HPV-16 E6 expression was shown to increase mTOR1 activity through the upstream activation of mTOR2 and 3-phosphoinositide-dependent kinase 1 (PDK1), leading to the activation of AKT [232]. Furthermore, HPV-16 E6 expression was shown to sustain the activation of receptor protein tyrosine kinases, including epidermal growth factor receptor (EGFR), insulin receptor beta, and insulin-like growth factor receptor beta, mediated via the signalling adaptor protein Growth Factor Receptor-Bound protein 2 (GRB2), which is upstream of the PI3K/AKT pathway [233].

Further, the nuclear accumulation of β-catenin has been shown to be associated with E6 in activation of the Wnt pathway. The mechanism is dependent on E6’s ability to interact with E6AP and independent of E6’s ability to target p53 for degradation or to bind to the PDZ-containing E6 targets [234]. Another mechanistic explanation for E6’s activation of Wnt pathways was shown to be through the downregulation of the seven in absentia homologue (Siah-1), which is involved in the proteasomal degradation of β-catenin [235]. While mice expressing wild-type E6 under the Keratin 14 promoter (K14E6 mice) showed enhanced nuclear accumulation of β-catenin and the accumulation of cellular β-catenin-responsive genes, mice expressing E6 lacking the PDZ-binding domain (K14E6∆PDZ) did not, indicating that E6 activation of Wnt signalling is, in part, PBM-dependent [236].

HPV-16 E6 has also been shown to activate the Notch pathway by interacting with NFX1-123 and increasing its expression, which, in turn, increases the Notch-1 mRNA levels in E6-expressing cells [237]. The modulation of Notch-1 by E6 was further shown to be mediated via presenilin-1 in mouse and human primary cell lines expressing HPV-16 E6 [238], while the expression of HPV-16 E6 prevents the early fate commitment of human keratinocytes towards differentiation and promotes cell proliferation at high cell densities through a combined inactivation of p53 and Notch-1 [239]. Furthermore, studies on cutaneous papillomavirus E6 oncoproteins have shown that E6 represses Notch signalling by association with MAML-1, a coactivator and effector of Notch-induced transcription, thereby delaying keratinocyte differentiation [240,241,242,243]. It is intriguing to note that E6 proteins from cutaneous HPV types target MAML through an LXXLL motif, while E6 proteins from mucosal HPV types target E6AP through the same mechanism. These interactions seem to have arisen early in the evolution of these viruses and to be related to their respective tropism for the mucosal or cutaneous epithelium, rendering the cellular environment amenable for viral replication [41,240,241,243]. These pathways are the key to cellular growth, proliferation and differentiation; thus, the continued expression of E6 and its targeting of these pathways forms a milieu where the perturbation of these signals overrides the normal homeostatic pathways and pushes the cell towards a carcinogenic phenotype.

### 2.4. Enabling Replicative Immortality

Normal cells have finite lifespan and can divide a limited number of times, known as the Hayflick limit. Molecularly, this limit to division is due to the shortening of telomeres at the ends of chromosomes, which can then no longer replicate, leading to signalling to activate the cellular apoptotic programme. To allow proper replication and cell division, the cellular telomerase enzyme maintains the length of the telomeres of the chromosomes and is tightly controlled in normal cells [244,245], and this is one of the main dysregulated activities in cancer cells. In HPV-mediated cancers, high-risk E6 proteins can activate telomerase in association with E6AP, and this was shown to be critical for immortalization [246,247]. The activation of hTERT transcription is driven by the c-Myc oncogene forming a heterodimer, c-Myc/Max, that binds to E-box sequences in the promoter of hTERT [248,249,250]. Although upstream stimulatory factors (USF1 and USF2) also bind to the same sites to disrupt the binding of c-Myc/Max, the expression of E6 downregulates the binding of USF1/2 at the promotor, further activating hTERT transcription [251]. Furthermore, GC-rich sequences found throughout the hTERT promoter, flanked by E-boxes, can enhance the activation of hTERT expression through SP1 transcription factors, and E6 has been shown to activate the SP1-dependent transcriptional activation of hTERT [249,252]. In addition to transcriptional activation of the hTERT promoter, the acetylation of histones at the hTERT promoter also enhances the telomerase expression. The continued passage of E6-expressing cells was shown to enhance this acetylation, whereas the knockdown of E6AP was shown to decrease the effect [253,254]. Thus, E6 in association with E6AP can activate the transcriptional regulation of hTERT, both through activators and repressors, as well as through epigenetic regulation. Furthermore, NFX-91, another transcriptional repressor, originally identified in a yeast two-hybrid screen with HPV-16 E6 and E6AP, was found to bind to a X1 box sequence in the hTERT promoter with co-repressor mSin3a, leading to histone deacetylase activity to deactivate hTERT expression. In HPV-16 E6-expressing cells, the levels of NFX1-91 are reduced through ubiquitin-mediated degradation by the E6/E6AP complex [254,255]. Yet another mechanism of activation of hTERT expression in E6-expressing cells is the expression of the splice variant of NFX1-123, which binds to the cytoplasmic poly(A)-binding proteins via a PAM2 motif and interacts with the poly(A) tail of mRNA, enhancing transcript shuttling via the nuclear-cytoplasmic route, recruitment of translational machinery and stabilization of mRNA, ultimately increasing the expression of hTERT in E6-expressing cells [256]. Thus, the expression of high-risk E6 proteins induces cellular changes that can lead to the replicative immortality of HPV-infected cells via multiple mechanisms involving transcriptional, post transcriptional, epigenetic and post-translational mechanisms.

### 2.5. Activating Invasion and Metastasis

The activation of invasive growth signals in cancer cells leads them to invade the surrounding tissue and the circulatory system, ultimately disseminating the cancer cells to distant anatomical sites. The regulation of this complex program often requires mechanisms involving a developmental program referred to as epithelial–mesenchymal transition (EMT) [1]. HPV oncoproteins E6 and E7 have been shown to activate EMT-inducing transcriptional factors—Slug, Twizt, ZEB1 and ZEB2—resulting in an increase in mesenchymal markers such as N-cadherin, fibronectin and vimentin and a decrease in epithelial cell markers like E-cadherin [257,258]. Furthermore, matrix metalloproteases (MMPs)—enzymes that are zinc-dependent endopeptidases playing crucial roles in various physiological processes, including tissue remodelling, organ development and the regulation of inflammatory processes—have been shown to be modulated in many cancers, including HPV-mediated cancer [259]. In the development and progression of HPV-mediated cancers, various members of the MMP family, including MMP-2, MMP-9 or MT1-MMP, have been shown to be upregulated both at the RNA and protein level in high-grade CIN, compared with normal cervix or low-grade CIN. Furthermore, the upregulation of MMP1, MT1-MMP, MMP2 and MMP9 has been shown to be due to the expression of HPV E6 and E7 oncoproteins in various cellular models of cervical cancer [260,261,262,263,264]. Mechanistically, the activation of AKT signalling by high-risk E7 has been linked to the downstream activation of transcription factors leading to *mmp* gene expression [260,265,266]. AKT activation has been further linked to CKII phosphorylation of high-risk E7, leading to the secretion of MMP1 and MMP13 associated with an invasive phenotype of HPV-18-positive C4-1 cervical cancer cells [267].

#### Role of E6 PDZ Binding Motif (PBM) and Polarity Deregulation

One of the unique features of high-risk E6 proteins is the presence of a PDZ (Post Synaptic Density 95 (PSD95), Discs Large (Dlg) and Zona Occludens 1 (ZO-1))-binding motif (PBM) (X-(S/T)-X-(V/I/L)-COOH) in the C-terminus, which is absent from E6 proteins of the low-risk HPV types [268,269]. The PBM is involved in binding to cellular proteins that have PDZ domains [270]. Over 20 different PDZ domain-containing proteins have been identified as targets of the high-risk E6 proteins [271]; amongst them are discs of large tumour suppressor (hDlg) [268,269], scribble tumour suppressor (hScrib) [272] and Membrane-associated guanylate kinase inverted 1 (MAGI-1) [273,274]. hScrib is involved in epithelial tight junctions and mediates the adhesion of basal cells to the extra-cellular matrix (ECM). Similarly, hDlg is involved in epithelial tight junctions, cell-to-cell junctions and epithelial polarity, while MAGI-1 has been suggested to colocalize with components of adherens junctions and tight junctions, and its expression probably promotes the assembly of macromolecular junctional complexes. hDlg, hScrib and MAGI-1 are tumour suppressors; the loss of these proteins facilitates cancer formation (reviewed in [275,276]), and all high-risk E6 proteins target them for proteasome-mediated degradation [272,277,278], most likely leading to the loss of cell polarity and facilitating tumour formation [279,280]. The affinities of interaction between different E6 PBMs and PDZ proteins are diverse, with single amino acid changes in the PBM switching the degree of preferred interaction, for example, HPV-16 E6 PBM (-ETQL) preferentially interacts with hScrib and HPV-18 E6 PBM (-ETQV) with Dlg [281]. Furthermore, the extent of promiscuity of the E6 PBM in interacting with several PDZ proteins has been shown to have strong correlation with the degree of carcinogenicity of the HPV type in cervical cancer [43]. Indeed, the degree of interaction with hScrib and another member of the apico-basal polarity (ABP) core component—the tight junction (TJ) protein ZO-2—is highly associated with a stronger cancer association (HPV-16, -18, -31, -35 and -51), suggesting that perturbation of the Scrib ABP complex is one of the crucial steps towards malignant transformation driven by E6 oncoprotein [282]. Cellular polarity deregulation in context of the viral life cycle also seems indispensable, as viruses defective in binding to polarity proteins are dampened in their ability to produce viral progeny and have deregulated viral genome maintenance, leading to a higher likelihood of the genome integrating into host DNA [283,284,285], thereby inducing deregulated expression of HPV oncoproteins and their carcinogenic orchestrations.

As discussed earlier, loss of PBM or perturbation of polarity protein targeting has been shown to induce integration of viral DNA into the host chromosome, the reason for which is yet unknown, however, it could potentially be a byproduct of a mishap in cell division, trying to uncouple cell polarity regulation in a proliferating cell. Asymmetric cell division of the basal cell with strict control of ABP regulation, mitotic spindle orientation and proper formation of cell-cell junctions are characteristic features of normal epithelial differentiation [286,287]. However, the E7-induced re-entry into cell cycle leading to aberrant proliferation, together with deregulation of Scrib and Par polarity complexes by HPV-E6 in the expanding population of infected cells in the mid-epithelial layer, potentially leads to enhanced symmetrical cell division, as expression of E7 alone has been suggested to induce aberration in spindle pole formation [288,289] and disruption of polarity complexes perturbs mitotic spindle orientation [290,291]. Taken together, HPV E6 and E7 by modulating EMT, extra-cellular matrix proteins, and cellular polarity regulators enhance cell plasticity in attaining invasive and potentially metastatic phenotypes.

### 2.6. Inducing Angiogenesis

Normal tissues and cells are continually supplied with nutrients and oxygen together with evacuation of metabolic waste and carbon dioxide. The vascular system finely works this out with proper morphogenesis and control throughout embryogenesis. After proper development, vasculogenesis and angiogenesis remain largely in a quiescent state unless reactivated by wound heading mechanisms or female reproductive cycling, but, again, only briefly. Tumours, like normal cells, also require the ‘services’ of the vascular system and often activate otherwise quiescent angiogenesis mechanisms to help sustain expanding neoplastic growths via an ‘angiogenic switch’ [292]. The angiogenic switch is tightly regulated and is often the result of countervailing activators or inhibitors, such as vascular endothelial growth factor-A (VEGF-A) and thrombospondin-1 (TSP-1), respectively. High-risk E6 and E7 oncoproteins have been implicated in modulating these regulators to induce angiogenesis via targeting p53 and pRB tumour suppressors. Inactivation of p53 by high-risk E6, also downregulates angiogenic inhibitors, TSP-1 and maspin, while upregulating VEGF-inducing angiogenesis [293,294,295,296,297]. VEGF is negatively regulated by p53 through HIF-1α and, as p53 is inactivated by E6, VEGF is induced to promote angiogenesis; however, VEGF can also be activated by p53-independent mechanisms through Sp1 transcription [298]. Furthermore, activation of AP1-dependent transcription by E7 can also induce VEGF through its AP1 binding site [299,300]. Additionally, RRM2-dependent induction of angiogenesis via ROS-ERK1/2-HIF-1α-VEGF has also been shown to be mediated via upregulation of RRM2 by E7 [301], and high-risk HPV-E7-dependent upregulation of hTERT has also been associated with upregulation of VEGF, potentially contributing to VEGF dependent angiogenesis [302].

### 2.7. Deregulating Cellular Energetics and Metabolism

Evading cellular growth control pathways leads to uncontrolled cellular proliferation and thus also imposes a substantial requirement for cellular energetics and metabolism to adjust to respond to the increasing cell numbers. Normal cells under aerobic conditions metabolize glucose to pyruvate via glycolysis and to carbon dioxide in mitochondria, while in anaerobic conditions glycolysis is more favoured, slowing down much of the oxidative metabolism of pyruvate; however, cancer cells can switch glucose metabolism chiefly to glycolysis even in the presence of oxygen, a process known as ‘aerobic glycolysis’ [303]. One mechanism of this preference for aerobic glycolysis in HPV-mediated cancer could be due to the expression of high-risk E7. Using mammalian cells expressing HPV-16 E7, Zwerschke et al. showed that E7 expression increases the intracellular concentrations of phosphoenolpyruvate (PEP) and fructose 1,6-bisphosphate (FEP) metabolites, upstream of the glycolytic enzyme type M2 (M2-PK). M2-PK occurs in a tetrameric form with a high affinity for PEP and a dimeric form with low affinity for PEP. While FEP induces the reassociation of the dimeric to tetrameric form of M2-PK, expression of E7 shifts this equilibrium to the dimeric state, although there is a significant increase in FEP levels in E7-expressing cells, thus leading to aerobic glycolysis these cells [304]. On the other hand, binding of high-risk E6 oncoprotein to Sorting Nexin 27 (SNX27), an important regulator of the endosomal retromer complex, has been shown to upregulate glucose uptake by cancer cells by modulating the expression of GLUT1 [305], leading to increased glycolytic flux in cervical cancer cells. While, switching to aerobic glycolysis from oxidative phosphorylation is less likely to be a binary switch mechanism, cancer cells are rather efficient in continuing to use oxidative phosphorylation in addition to incorporating variable rates of glycolysis, which again might be dependent upon cancer cell location in different subregions within a tumour and its microenvironment [2]. For instance, in HNSCC, HPV-positive tumours were shown to display increased levels of oxidative phosphorylation and higher rates of aerobic glycolysis in the tumour core in contrast to HPV-negative tumours. This differential metabolism was linked to increased centrally localized staining of glucose transporter 1 (GLUT1), lactate dehydrogenase B, monocarboxylate transporter 1 and cyclooxygenase 5B in HPV-positive tumours compared with more peripheral staining in HPV-negative tumours [306]. Taken together, these data suggest that in HPV-positive tumours expression of E6 and E7 seem to be cooperatively driving aerobic glycolysis in the tumour to sustain induced proliferation. Moreover, expression of HPV-16 E6 can activate mTORC1 signalling and increase protein synthesis both under normal and limited growth factor conditions [232,233]. This would increase the need for building blocks for sustained proliferation, potentially more so in an environment such as a poorly vascularized tumour core, which presumably is more restricted for nutrients and energy sources than the tumour periphery.

### 2.8. Genome Instability and the Consequent Mutation of Hallmark-Enabling Genes

Despite the fact that expression of high-risk oncogenes in primary human keratinocytes can cause cellular immortalization, with the cells exhibiting many characteristics of premalignant lesions, these cells do not form tumours when injected into nude mice. Additional oncogenic events are necessary for malignant progression to occur, such as expression of oncogenes like *ras* or *fos*, or accumulation of oncogenic mutations over prolonged passaging in culture [307,308,309,310]. A major contributor to genomic instability has been suggested to be expression of high-risk HPV E7 [311]. HPV-16 E7 has been shown to induce centrosomal duplication errors, leading to multipolar mitoses, chromosome mis-segregation and aneuploidy, independently of its RB-inactivating activity [312]. Further, HPV-16 E7 was shown to associate with the centrosomal regulator, γ-tubulin, altering its recruitment to the centrosome in HPV-16 E7-expressing cells, suggesting a role for E7 in abnormal centrosomal amplification and disruption of centrosome homeostasis [313]. Abnormal centriole multiplication was also shown to correlate with up-regulation of Polo-like kinase 4 in HPV-16 E7-expressing cells [314].

Furthermore, HPV-16 E7 expression was shown to induce the delocalization of dynein from mitotic spindles [315]. It was also shown that the interaction of HPV-16 E7 with nuclear mitotic apparatus protein 1 (NuMA) corelated with induction of defects in chromosome alignment during prometaphase, irrespective of normal centrosome numbers, indicating that disruption of the NuMA/dynein network results in mitotic errors that would make an infected cells more prone to accumulation of aneuploidy, even in the absence of supernumerary centrosomes [316].

In addition, high-risk E7 targets ATM/ATR DNA damage response pathways: HPV-31 E7 was shown to bind ATM, inducing its phosphorylation and activating Chk2 [317], while HPV-18 E7 was shown to induce increased levels of phosphorylated ATM and the downstream kinases Chk1, Chk2 and JNKs (c-jun N-terminal kinases) [318]. In addition, high-risk E7 was shown to target claspin, a key regulator of the ATR-Chk1 pathway that is activated in response to replications stress. HPV-16 E7 also attenuates mitotic checkpoint control by upregulating cellular factors involved in destabilization of claspin—a positive regulator of the mitotic checkpoint; this activity is primarily dependent E7’s inactivation of pRB, as most of the factors involved in turnover of claspin are regulated by E2F transcription factors [319]. Thus, E7-induced accelerated degradation of claspin in G2/M leads cells to initiate checkpoint recovery, even in the presence of DNA damage that could potentially lead to genomic instability [319].

Furthermore, HPV oncogenes hinder the homologous-recombination repair pathway, where HPV E7 impairs RAD51 localization to transient lesions (double strand breaks [DSB]), impairing DSB repair, and contributing to genomic instability [320]. More recently, HPV E7 has been shown to hijack the E3 ubiquitin ligase RNF168, which is critical to proper DSB repair, in order to promote the viral replication cycle. This interaction perturbs cellular DSB signalling, leading to disruption of host chromatin response to DNA breaks and promoting genomic instability that drives oncogenesis [321].

An initial event in the induction of genomic instability in HPV infection, and probably responsible for cases of co-existing episomal and integrated HPV DNA, is the upsetting possibility of ‘onion skin’ type of HPV DNA replication, caused by activation of the viral origin of replication multiple times within a single cell cycle, leading to single- or double-strand breaks and recruitment of the DNA repair machinery, thus potentially promoting chromosomal defects [322]. In addition to the genomic instability induced by HPV proteins, HPV-mediated cancers are frequently found to have somatic mutations, including those driven by apolipoprotein B mRNA editing catalytic polypeptide-like (APOBEC), copy number variations and large chromosomal rearrangements. Some of the key genes often mutated in HPV-associated cancers are in PIK3CA (phosphatidylinositol-4,5-bisphosphate 3-kinase catalytic subunit alpha), PTEN (phosphatase and tensin homologue), HLA-A and HLA-B (human leukocyte antigen A and B), TGFβ (transforming growth factor beta), and components of the NOTCH1 and RAS/EGFR/ERK pathways; in contrast mutations are very rarely found in p53 and pRB [13,21]. Additional mutations in these genes that regulate several homeostatic processes further augment carcinogenic progression, however, how these often-detected somatic mutation contribute to HPV-mediated carcinogenesis and how HPV-infection ultimately leads to these mutational signatures is not fully understood.

#### 2.8.1. HPV E7 and Epigenetic Reprograming

Alterations in DNA methylation are associated with a number of human diseases and are one of the hallmarks of cancer. The HPV-16 E7 oncoprotein has been shown to bind directly to DNMT1 and stimulate its methyltransferase activity [323]. Furthermore, HPV E7 proteins have been shown to interact with both HATs and HDACs. HPV-16 E7 is known to interact with p300/CBP-associated factor (PCAF) histone acetyltransferase [324] and this activity can contribute to downregulation of IL-8, which might, in turn, contribute to the ability of infected cells to avoid the host immune response [325].

HPV E7 has also been shown to interact with HDAC1 and HDAC2 through the Mi2β protein [326]: both Mi2β and HDAC1/HDAC2 are components of the NuRD chromatin remodelling complex. This interaction has been demonstrated to modulate histone modification and transcription of cellular genes relevant to cell cycle deregulation [327] or immune evasion [328]. HPV-16 E7 interacts with interferon regulatory factor-1 (IRF-1), which activates the IFN-β gene; however, by recruiting HDAC to abrogate the transactivation function of IFR-1, E7 has been suggested to suppress a cellular immune response to HPV infection [328]. Furthermore, the association of HPV-31 E7 and HDACs in differentiated cells is involved in activation of E2F2 gene transcription, facilitating HPV-31 replication [329]. HPV E7 has also been shown to enhance HIF-1α-dependent transcription by inducing dissociation of HDAC1, HDAC4 and HDAC7 from HIF-1α, which might also contribute to tumour angiogenesis [330].

HPV E7 has also been shown to induce expression of histone H3 lysine 27 demethylase, KDM6A and KDM6B, enzymes responsible for H3K27me3 demethylation [331]. Further, KDM6B induction mediates increased expression of the cervical cancer biomarker p16^INK4A^. Higher expression of p16^INK4A^ caused by HPV-16 E7 mediated KDM6D upregulation represents an E7-triggered oncogene-induced senescence (OIS) response. This response, as RAS/RAF cause KDM6B upregulation, leads to de-repression of p16^INK4A^ transcription, followed by inhibition of CDK4/6 activity and inhibition of pRB phosphorylation. The ultimate effect is G1 cell cycle arrest and senescence; however, HPVs have evolved to target pRB for ubiquitin-dependent proteasomal degradation, which is why p16^INK4A^ upregulation in HPV-positive cancer cells does not inhibit proliferation [332,333,334]. Further, KDM6A- and KDM6B-responsive Homeobox (HOX) genes are expressed at significantly higher levels, suggesting that ectopic expression of HPV-16 E7 results in reprogramming of host epithelial cells [331]. Furthermore, increased KDM6A in response to high-risk HPV E7 expression was shown to cause de-repression of the cell cycle and DNA replication inhibitor p21^CIP1^ and this activity was shown to be required in high-risk E7 expressing cells for p21^CIP1^’s ability to inhibit DNA replication through PCNA binding [335].

The polycomb group of proteins forms polycomb repressive complexes (PRC) that repress gene transcription [336]. PRC2, for instance, silences genes by trimethylating the lysine residue 27 of histone H3, while PRC1 binds to H3K27me3-marked chromatin and further silences gene expression by monoubiquitinating lysine K119 of histone H2A. HPV-16 E7 associates the E2F6 factor with multiple polycomb protein, including BMI1, PCGF2 (MEL-18), CBX4 (hPC2), RING1, MGA, and L3MBTL2, to abrogate the repressive activity of E2F6 on its target genes [159]. HPV-16 E7 has also been shown to induce expression of H3K27 histone methyltransferase EZH2 (enzymatic component of PRC2), enhancing PRC4 complex formation [337], which has been demonstrated to cause histone H1K26 deacetylation and methylation [338].

#### 2.8.2. Modulation of MicroRNAs by HPV E7

Among many other factors, microRNAs (miRNAs) are also known to regulate the expression and activities of cellular proteins by acting as post-transcriptional regulators of gene expression [339]. These are small RNA molecules (18–25 nucleotides), transcribed by RNA polymerase II. HPV E7 has been shown to downregulate miRNA203, which is normally expressed at higher levels in differentiating cells to downregulate the p63 family of transcription factors, thereby inhibiting cell proliferation. However, E7-mediated downregulation of miRNA203 appears to be necessary for genome amplification and productive replication in differentiating cells [340]. A number of other microRNAs are also modulated by expression of E6 and E7 oncoproteins, singly or in combination. For instance, miR-33b-3p, -542-3p and -33-3p—are upregulated and miR-193b-3p is downregulated in HPV-16 E6/E7 expressing HFKs. Specifically, upregulation of miR-16-2-3p and downregulation of miR-197-3p and -1249 in HPV-16 E6/E7 expressing HFKs is driven by E7 expression [341]. These microRNAs, together with others that are modulated by HPV oncoproteins, seem to affect several of the cellular signalling pathways including p38 MAPK signalling, G1/S checkpoint regulation, and ATM signalling, contributing towards rewiring of cellular regulatory pathways to oncogenic transformation [341,342].

### 2.9. Avoiding Immune Destruction

Another facet of the development and progression of tumours and cancers is compromised immune detection. Immunological surveillance for virus-infected cells is constantly monitored by our ever-alert immune system, to resist and eradicate the formation and progression of incipient neoplasias. However, high risk-HPV oncoproteins play a significant role in perturbing this surveillance system by perturbing the expression and trafficking of several immune receptors and mediators, essential for detection and targeting of virus-infected cells. Foreign antigen presentation on major histocompatibility complex (MHC) class I molecules is important for T-cell recognition of virus-infected cells, but HPV E5 proteins from several HPV types (16, 2a and 83) have been demonstrated to specifically downregulate this class of surface molecules (HLA-A and HLA-B) [343,344]. Mechanistically, this has been linked to interaction of HPV-16 E5 with MHC class I through E5’s first helical transmembrane domain (TM1), and to E5’s location in Golgi/ER, possibly interfering with the trafficking of MHC molecules to the cell surface, as dislocation of E5 from Golgi/ER leads to the abrogation of MHC I downregulation [345,346]. Furthermore, a ternary complex with calnexin, HPV-16 E5 and the heavy chain of HLA-I, via the first hydrophobic region of the E5 protein, has been suggested to be responsible for retention of HLA-I molecules in the ER of the cells [347]. In addition, maturation of MHC class II molecules in human foreskin keratinocytes upon interferon gamma treatment has also been suggested to be perturbed by expression of HPV-16 E5. This function was demonstrated to occur through HPV-16 E5’s inhibition of endosome acidification, which prevents the breakdown of invariant chain and blocks formation of peptide-loaded MHC class II dimers, leading to decreased surface expression of MHC class II molecules, thus disrupting antigen presentation to effector T-cells [348]. Similarly, HPV-16 E5 perturbs the expression of CD1d, which is yet another surface receptor important for immune surveillance by natural killer cells. Upon HPV-16 E5 expression in both C33A cancer cell line and normal human keratinocytes, CD1d levels were downregulated via proteasomal degradation, with the inhibition of calnexin-related CD1d trafficking potentially protecting HPV-infected cells from immunological surveillance [349]. Interestingly, a recent study in head-and-neck squamous cell carcinoma (HNSCC) has shown evidence of E5-mediated immune evasion by suppressing the MHC complex and interfering with antigen presentation in both murine models and patients’ tumours. These tumours, expressing HPV-16 E5 were resistant to anti-PD-1/PD-L1 immunotherapy, however, use of the antiviral E5 inhibitor, rimantadine, improved the response of this checkpoint blockade immunotherapy, suggesting that HPV-E5 might be evading the T-cell response by abrogating its effector mechanism via PD-L1 expression [350]. Interferons are one of the primary immune defence mechanisms to viral infection and signal antiviral strategies in nearby cells. However, HPV-16 E5 can dysregulate interferon (IFN) signalling by suppressing STAT1, leading to suppression of downstream IFN-stimulated genes (ISGs) in human keratinocytes. Mechanistically, abrogated ISG expression was shown to depend upon E5-induced EGFR signalling, which in the absence of E5 would otherwise have led to TGFBR2 signalling, resulting in increased production of IFN [351].

High-risk E6 and E7 also contribute to modulating the immune response to infection. HPV-16 E6 interaction with IFN regulatory factor 3 (IFR3) has been suggested to prevent the transactivation of IFN-beta expression [352]. In HPV-18 E6 expressing cells, JAK/STAT signalling has been shown to be impaired by affecting activation of non-receptor tyrosine kinase 2 via direct interaction with the kinase [353]. High-risk E6 proteins can also attenuate Retinoic acid-inducible gene I (RIG-I)-mediated signalling by promoting ubiquitination and degradation of TRIM25, an activator of RIG-I signalling, thus dampening type-I interferon production [354]. Furthermore, high-risk E6 proteins have also been demonstrated to inhibit transcription of kappa interferon (IFNκ), specific to keratinocytes, by methylation of its promoter, thereby attenuating activation of antiviral ISGs and pattern recognition receptors (PRRs) [355]. Host cell PRRs are major targets for immune evasion by HPV and HPV oncoproteins have been demonstrated to repress this innate immune response. High-risk HPV-18 E7 can directly bind to STING via the pRB binding motif, resulting in inhibition of the cGAS-STING pathway, involving recognition of foreign DNA [356]. Additional epigenetic mechanisms active in HPV-positive cancers, leading to suppression of the cGAS-STING pathway, have also been suggested, involving E7-induced upregulation of SUV39H1 methyltransferase and downregulation of histone demethylases KDM5B important for cGAS-STING expression [357,358]. Furthermore, double-stranded DNA sensor Toll-like receptor 9 is also efficiently silenced by recruitment of KDM5B and histone deacetylase HDAC1 to the TLR9 promoter, abrogating type-1 interferon secretions [359]. Taken together, these studies show that the efficient action of HPV oncoproteins in evading immune responses allows HPVs to avoid destruction and is one of the major steps towards persistent and/or chronic infection potentially leading to carcinogenesis.

### 2.10. Tumour-Promoting Immune Cell Infiltration (Inflammation)

Cellular, innate, and adaptive immune responses, with an array of secreted cytokines, play a significant role in progression of both infection and cancer. Inflammation is a host defence strategy against foreign agents, actively mediated to the site of breach (or stimulus) and involves the release of cytokines and mediators that act to facilitate recruitment of effector cells to the site of injury. In most instances, the inflammatory process is disabled upon clearance of the stimulus, however, if it persists, inflammation can tend to become chronic and can lead to cancer. Expression of high-risk HPV oncoproteins can induce an inflammatory response by upregulating pro-inflammatory cytokines and chemokines [360,361,362]. Very often tumours are infiltrated by a variety of cell types called infiltrating immune cells or IIC [2], and increase in IIC at the HPV-associated lesions correlates with high-grade lesions [362,363,364]. Using an HPV-16 transgenic mice model, it has been shown that macrophage recruitment at the lesion site was dependent on CC chemokine ligand-2 (CCL2) and its receptor CCR2. B-cell responses in this case were shown to aggravate the condition to chronic inflammation and to promote tumour progression due to extracellular matrix deposited by E6/E7 expressing cells [365,366,367]. Another effect of the inflammatory response upon tumour progression is the associated DNA damage in the infected tissue. In high-grade HPV-positive cervical lesions, higher accumulation of mutagenic DNA lesion 8-nitroguanine was observed, caused by nitric oxide and reactive oxygen species produced by inflammatory cells [368]. Furthermore, Langerhans cells, the major antigen presenting cell population of the skin, were demonstrated to be reduced in numbers in HPV-associated lesions, potentially via down regulation of E-cadherin expression mediated by E6. In addition, Langerhans cells were also found to be unable to initiate T-cell responses when exposed to chimeric HPV-16 virus like particles via a mechanism dependent on phosphatidylinositol kinase-3 [369,370,371,372] suggesting that expression of HPV oncoproteins in the infected epithelium deregulates inflammatory responses in order to sustain viral persistence. There is a concomitant failure of IICs to eliminate virus-infected cells, thus aggravating the response to chronic inflammation and potentially leading to tumour progression. In addition, certain studies have also indicated that other sexually transmitted viral or bacterial pathogens, such as herpes simplex virus type 2 (HSV-2) or *Chalmydia trachomatis,* may well serve as cofactors for the development of the intense chronic inflammatory response leading to HPV-associated disease [373,374].

## 3. Conclusions

The expression of HPV E5, E6 and E7, taken together, results in the abrogation of multiple cellular homeostasis pathways or, often, in their re-direction towards growth and proliferation, immune evasion, differentiation delay, inhibited apoptosis, genome instability and, consequently, immortalization. Although the primary function of these proteins early in viral life cycle is simply to produce a cellular milieu conducive for viral replication. As with many cancers, HPV-mediated cancers are the result of deregulation of multiple cellular functions, ultimately and unfortunately leading to accumulation of mutations in the host genes, further unbalancing the normal homeostasis mechanisms and leading to cancer and malignancy. Excellent vaccines are the keys to prevent initial infection; however, as discussed earlier, for cases of persistent infection there is a likelihood of development of cancer and malignancy over several years, orchestrated by expression of E5, E6 and E7—the not-so-good, the bad and the ugly—in terms of their molecular *prognosis/outcome,* as discussed above. Despite a multitude of data regarding the mechanisms behind the carcinogenic effects of these viral oncoproteins, many mechanisms still remain to be uncovered (refer to review [375]), and specific therapeutic agents against HPV-mediated cancer have not yet been found. However, ongoing clinical trials of promising therapeutic agents against specific targets, and more studies further elucidating the mechanisms of carcinogenic orchestration by these oncoproteins, will eventually aid in the development of therapy against HPV-mediated disease and carcinogenesis. Furthermore, the unique insights that E5, E6 and E7 are providing into the underlying mechanisms of carcinogenesis will also have major implications for our overall understanding of cancer development in general.

## Figures and Tables

**Figure 1:**
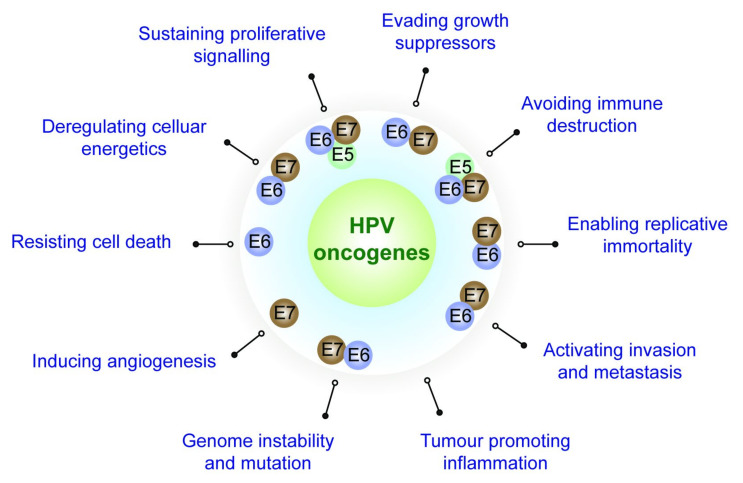
The hallmarks of cancer adapted from Hanahan and Weinberg 2016 [2]. A schematic illustrating eight distinct functional capabilities and two facilitators that are necessary conditions for the manifestation of malignant disease—cancer—and the expression of the HPV oncoproteins responsible for attaining these functions.
